# Supplementary material for: The Susceptibility of Chickens to Zika Virus: A Comprehensive Study on Age-Dependent Infection Dynamics and Host Responses
Source: Viruses. 2024 Apr 7;16(4):569. doi: 10.3390/v16040569 (PMC11054531; doi:10.3390/v16040569)
Supplement: Supplementary file 1 [file viruses-16-00569-s001.zip › viruses-2922790-supplementary.pdf]

Supplementary Material for Susceptibility of Chickens to Zika Virus: A Comprehensive Study on Age-Dependent Infection Dynamics and Host Responses by Nissly *et al.*

Supplementary Table 1: Primers used for gene expression experiments.

Gene	Forward Primer (5' to 3')	Reverse Primer (5' to 3')	Source
IFNα	CTTCCTCCAAGACAACGATTACAG	AGGAACCAGGCACGAGCTT	Kuchipudi *et al.* [1]
IFNβ	CCTCCAACACCTCTTCAACATG	TGGCGTGCGGTCAAT	Suzuki *et al.* [2]
18S	TGTGCCGCTAGAGGTGAAATT	TGGCAAATGCTTTCGCTTT	Kuchipudi *et al.* [1]
MDA5	AGGAGGACGACCACGATCTCT	CCACCTGTCTGGTCTGCATGT	Chhabra *et al.* [3]
IL1β	TGCTGGTTTCCATCTCGTATGT	CCCAGAGCGGCTATTCCA	Chhabra *et al.* [3]
OASL	ACATCCTCGCCATCATCGA	GCGGACTGGTGATGCTGACT	Barber *et al.* [4]

Supplementary Table S2: Primers and probe used for Zika virus RNA detection[1].

Name	Type	Sequence (5' to 3')
Zika-Dual-For	primer	ATATCGGACATGGCTTCGGA
Zika-IbH-Rev	primer	GTTCTTTTACAGACATATTGAGTGTC
Zika-Dual	FAM-labeled probe	TGCCCAACACAAGGTGAAGCCTACCT

[1] primers and probe were first described in Goebel *et al* [5].

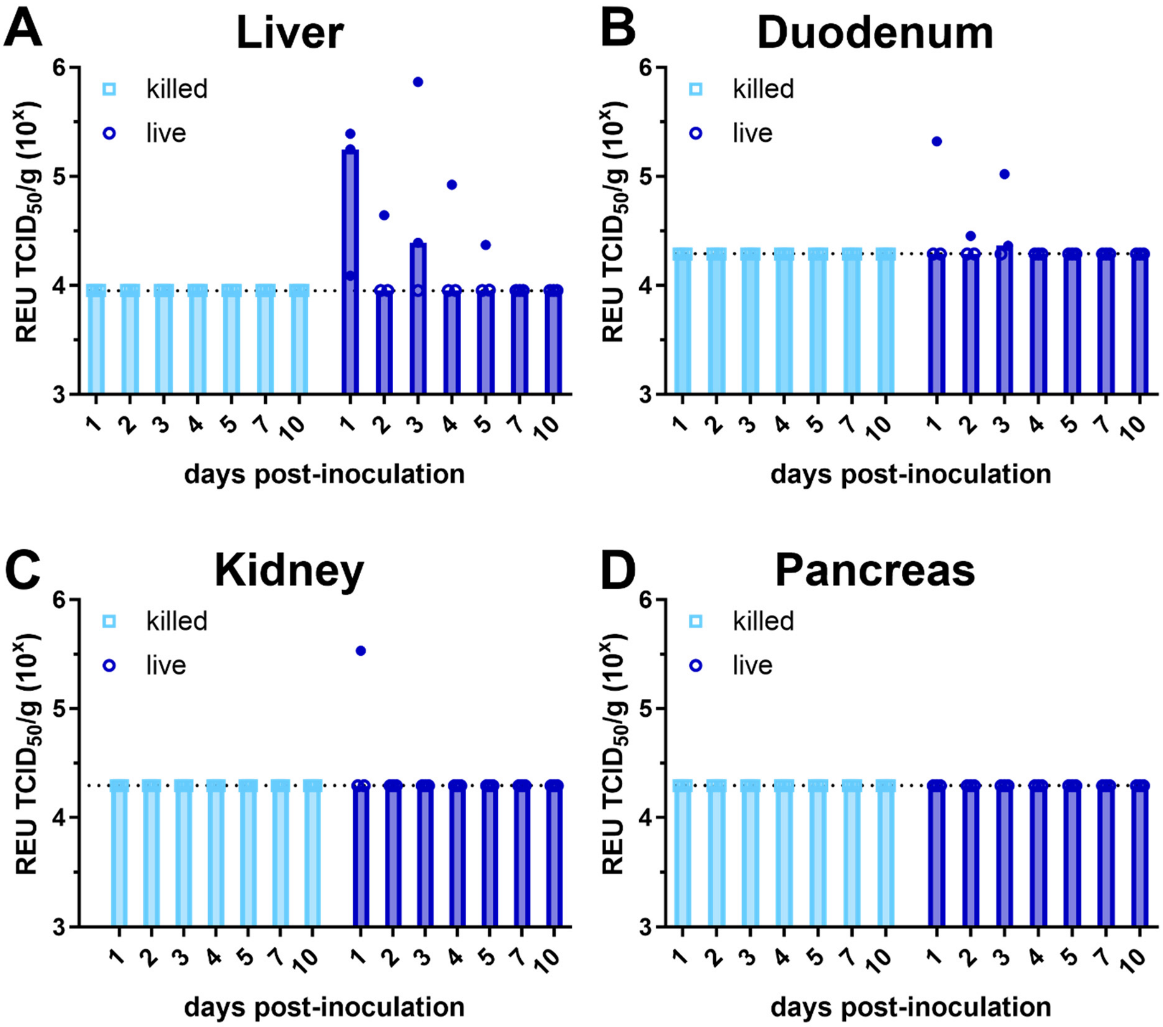

Supplementary Figure S1: Viral RNA detection in additional tissues of 1 day-old (1d.o.) chickens inoculated with live or killed Zika virus. Chickens were inoculated with 10^7 $TCID_{50}$ units of infectious Zika virus (ZIKV; live) or UV-inactivated ZIKV (killed), and blood and tissues were collected following euthanasia at seleted timepoints as described in Figure 4. ZIKV viral RNA quantified by real-time PCR relative equivalent units (REU) against a $TCID_{50}$ standard are shown. The median of animals per timepoint is shown by height of bar graph. Symbols represent measurements from individual animals in liver (A), duodenum (B), kidney (C), and pancreas (D). Open symbols represent individuals with undetectable viral RNA, at or below the limit of detection (LOD), which is shown as a dashed line. All specimens tested from killed inoculum group had undetectable viral RNA.

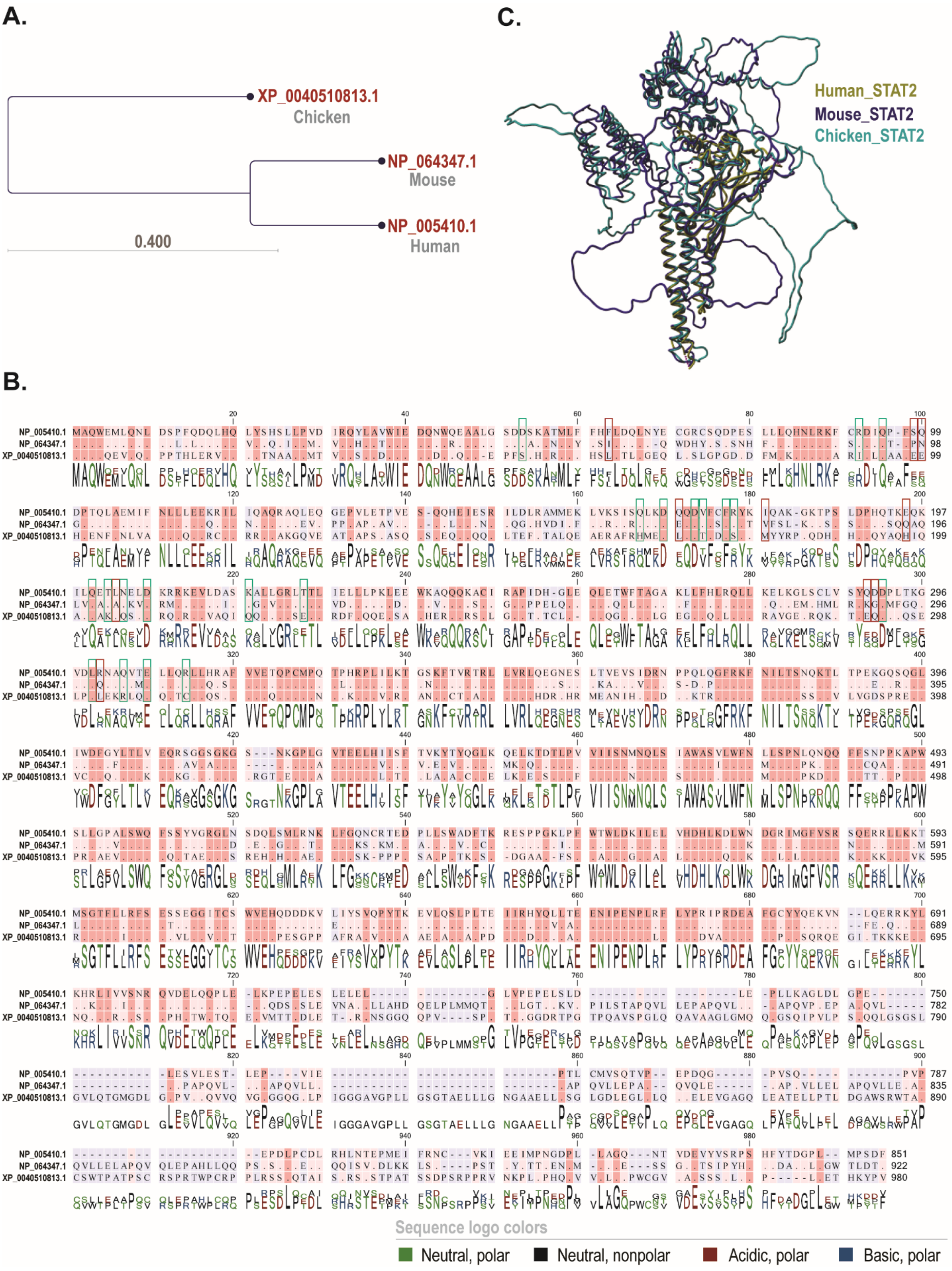

A.
XP_0040510813.1
Chicken
NP_064347.1
Mouse
NP_005410.1
Human
0.400
C.
Human_STAT2
Mouse_STAT2
Chicken_STAT2
B.
NP_005410.1
NP_064347.1
XP_0040510813.1
Sequence logo colors
Neutral, polar
Neutral, nonpolar
Acidic, polar
Basic, polar

Supplementary Figure S2: STAT2 protein comparison between chicken, human and mouse. (A) Maximum likelihood phylogeny analysis of STAT2 of human, mouse, and chicken was performed using the neighbor joining method (Kimura 80 nucleotide substitution model). (B) Multiple sequence alignment of amino acids. Boxed amino acids represent residues implicated in binding between Zika virus NS5 and human STAT2 [6]. Green boxes represent those binding amino acids with homology between human and mouse. Amino acids within the red boxes represent variation among the species. The coiled-coil domain (CCD) includes residues 131 through 319 using the alignment numbering (residues 140 to 315 of human STAT2). (C) Structural alignment of human (PDB ID: 6WCZ), mouse, and chicken STAT2. Mouse (UniProt ID: Q9WVL2) and chicken (UniProt ID: A0A3Q2U7J6) structures were generated with AlphaFold [7] and structural alignment was performed using YASARA (Version 23.12.24).

References

1. Kuchipudi, S.V.; Tellabati, M.; Sebastian, S.; Londt, B.Z.; Jansen, C.; Vervelde, L.; Brookes, S.M.; Brown, I.H.; Dunham, S.P.; Chang, K.-C. Highly pathogenic avian influenza virus infection in chickens but not ducks is associated with elevated host immune and pro-inflammatory responses. *Veterinary Research* **2014**, *45*, 118, doi:10.1186/s13567-014-0118-3.
2. Suzuki, K.; Okada, H.; Itoh, T.; Tada, T.; Mase, M.; Nakamura, K.; Kubo, M.; Tsukamoto, K. Association of increased pathogenicity of Asian H5N1 highly pathogenic avian influenza viruses in chickens with highly efficient viral replication accompanied by early destruction of innate immune responses. *J Virol* **2009**, *83*, 7475-7486, doi:10.1128/jvi.01434-08.
3. Chhabra, R.; Kuchipudi, S.V.; Chantrey, J.; Ganapathy, K. Pathogenicity and tissue tropism of infectious bronchitis virus is associated with elevated apoptosis and innate immune responses. *Virology* **2016**, *488*, 232-241, doi:doi.org/10.1016/j.virol.2015.11.011.
4. Barber, M.R.; Aldridge, J.R., Jr.; Fleming-Canepa, X.; Wang, Y.D.; Webster, R.G.; Magor, K.E. Identification of avian RIG-I responsive genes during influenza infection. *Molecular immunology* **2013**, *54*, 89-97, doi:10.1016/j.molimm.2012.10.038.
5. Goebel, S.; Snyder, B.; Sellati, T.; Saeed, M.; Ptak, R.; Murray, M.; Bostwick, R.; Rayner, J.; Koide, F.; Kalkeri, R. A sensitive virus yield assay for evaluation of antivirals against Zika Virus. *Journal of Virological Methods* **2016**, *238*, 13-20, doi:10.1016/j.jviromet.2016.09.015.
6. Wang, B.; Thurmond, S.; Zhou, K.; Sánchez-Aparicio, M.T.; Fang, J.; Lu, J.; Gao, L.; Ren, W.; Cui, Y.; Veit, E.C.; et al. Structural basis for STAT2 suppression by flavivirus NS5. *Nature Structural & Molecular Biology* **2020**, *27*, 875-885, doi:10.1038/s41594-020-0472-y.
7. Jumper, J.; Evans, R.; Pritzel, A.; Green, T.; Figurnov, M.; Ronneberger, O.; Tunyasuvunakool, K.; Bates, R.; Zidek, A.; Potapenko, A.; et al. Highly accurate protein structure prediction with AlphaFold. *Nature* **2021**, *596*, 583-589, doi:10.1038/s41586-021-03819-2.
